# Leith Fever Hospital

**Published:** 1893-05-06

**Authors:** 


### HOSPITAL CONSTRUCTION.

### LEITH FEVER HOSPITAL.

In examining the plans for this projected new hospital, one is struck with two things, first the peculiarly unsuitable nature of the site, and secondly the remarkable fascination which the plan of the ward blocks at Newcastle-on-Tyne appears to exercise on the minds of hospital committees.

The site in question is nearly square, and has a tolerably steep slope towards the north, on which side, and also on the north-east, it has a high railway embankment abutting on it. Just outside the north boundary, and between it and the railway embankment, is a stream running parallel with the former.

Thus it will be seen that in point of aspect the site is exactly the reverse of what it should be, and that, at its lowest part, where ventilation is most needed, the free movement of air is impeded by two embankments, and hygienic conditions are still further lowered by the presence of the stream referred to.

To utilise to the greatest advantage a site of this nature would be at best a difficult and thankless task; but we cannot help thinking that in this case, unless there are other and greater difficulties which do not appear on the plans before us, the architect has not done the best with the ground at his disposal. The five ward blocks are all placed with their long axes East and West instead of North and South, an arrangement which must clearly result in the obstruction of unlight to the greatest possible degree, besides providing that one side of every ward shall never get any direct sunlight at all. Though the site is at best of but limited size for so large a number of buildings, there is nothing apparent in the plans to warrant the assumption that there would have been any difficulty in placing the ward blocks North and South instead of East and West.

As to the ward blocks themselves, it is evident that they have been modelled on the lines of the City Hospital for Infectious Diseases at Newcastle-on-Tyne. Why this particular form of pavilion, with its many obvious defects, should have been so frequently copied it is hard to say, except that in so many cases the designing of fever hospitals is entrusted to the inexperienced hands of borough surveyors from short-sighted considerations of parsimony. These officials, who in many cases are not architects at all, but engineers, have to produce a plan which shall pass muster at Whitehall, and, not having the necessary time at their disposal to make themselves acquainted with the special needs of hospital arrangements, avail themselves, naturally enough, of ready-made examples. So it has happened that in many recent instances the Newcastle type has been reproduced and its defects blindly copied.

The pavilion in question consists of two wards, divided by a nurses' duty-room and lobby. At the entrance is a projecting wing containing a waiting-room, scullery, coal-shed, and w.c. The scullery ought not to be required at all if the nurses' room is properly equipped, and the waiting-room should be at the porter's lodge, between which and the wards there should be telephonic communication. On the other side of the pavilion are two projecting buildings, each being a ward for one bed. These wards communicate with the main ward, and are, therefore, practically useless as separation wards for noisy or offensive cases. At the ends of each of the main wards are two other projecting wings—one containing the bath-room, the other the closets. There are thus no less than seven projections from the main block, each serving more or less to intercept the free access of sunlight to the main wards, besides obviously making the building more costly in construction.

When we add that in placing the buildings on the ground—the mortuary, two fever wards, and the destructor are put within twenty feet of the boundary, the last-named being actually on the boundary—our readers will see what an ill-digested imperfect scheme is being contrived for dealing with the infectious diseases of Leith.

